# Antioxidants in the Fight Against Atherosclerosis: Is This a Dead End?

**DOI:** 10.1007/s11883-018-0737-7

**Published:** 2018-05-21

**Authors:** Paola Toledo-Ibelles, Jaime Mas-Oliva

**Affiliations:** Instituto de Fisiología Celular, Universidad Nacional Autónoma de México, Mexico City, Mexico

**Keywords:** Atherosclerosis, Cardiovascular disease, Antioxidant therapy, Redox reactions, Standardization factors

## Abstract

**Purpose of Review:**

The purpose of this review is to focus on the outcome of recent antioxidant interventions using synthetic and naturally occurring molecules established as adjuvant strategies to lipid-lowering or anti-inflammatory therapies designed to reduce the risk of cardiovascular disease.

**Recent Findings:**

To date, accumulated evidence regarding oxidation as a pro-atherogenic factor indicates that redox biochemical events involved in atherogenesis are indeed a very attractive target for the management of cardiovascular disease in the clinic. Nevertheless, although evidence indicates that redox reactions are important in the initiation and progression of atherosclerosis, oxidation with a pro-atherogenic context does not eliminate the fact that oxidation participates in many cases as an essential messenger of important cellular signaling pathways. Therefore, disease management and therapeutic goals require not only high-precision and high-sensitivity methods to detect in plasma very low amounts of reducing and oxidizing molecules but also a much better understanding of the normal processes and metabolic pathways influenced and/or controlled by oxidative stress. As several methodologies have been specifically described for the quantification of the total antioxidant capacity and the oxidation state of diverse biological systems, a successful way to carefully study how redox reactions influence atherosclerosis can be achieved.

**Summary:**

Since there is still a lack of standardization with many of these methods, clinical trials studying antioxidant capacity have been difficult to compare and therefore difficult to use in order to reach a conclusion. We believe a comprehensive analysis of new knowledge and its relationship with the presence of plasma antioxidants and their reducing capacity will undoubtedly open new ways to understand and develop new therapeutic pathways in the fight not only against atherosclerosis but also against other degenerative diseases.

## Oxidative Stress and Atherosclerosis

Cardiovascular diseases (CVDs) are nowadays considered the clinical complications with the greatest impact on mortality in the western world. According to WHO’s most recent records, CVDs were responsible for a total of 46% of deaths due to non-communicable diseases in 2012 [1]. Among CVDs, coronary heart disease and stroke are the ones with the greatest impact since in addition to causing a high frequency of deaths, they occur at ages that are still far from the maximum life expectancy as rated by WHO [2, 3]. These diseases are the clinical manifestation of the pathophysiological process known as atherosclerosis, related to inflammation and the accumulation in the blood vessels of the ketone and hydroxide forms of lipids derived from the non-enzymatic oxidation of cholesterol and polyunsaturated fatty acids (PUFA) [4, 5]. It has been proposed that these lipids originate from low-density lipoproteins (LDLs), which in their oxidized form (oxLDL) are taken up by receptors such as LOX-1, SR-A, and SR-B. Unlike the receptor that recognizes native low-density particles (LDLR), these are not regulated by intracellular cholesterol levels and allow an excessive increase of cholesterol inside the cell generating the so-called macrophage-derived foam cells [5].

Following pioneer studies carried out by Goldstein and Brown who first proposed a binding site on macrophages for chemically modified LDL uptake such as acetylated and maleylated LDL [6]; in the 1980s and the 1990s, classical studies by J.L. Witztum defined that an important LDL modification is associated with the oxidation of LDL particles [7]. By incubating LDL with cultured endothelial cells or smooth muscle cells, they showed that the newly created oxidized forms are rapidly internalized in a saturable manner.

Currently, the most widely accepted hypothesis regarding atherogenesis proposes that atherosclerosis initiates its development with LDL entrance from the bloodstream into the subendothelial space, between the tunica intima and media, where cellular metabolism fosters its oxidation, phagocytosis by macrophages and their consequent overloading of intracellular lipids [8, 9]. Moreover, oxLDLs act as a chemotactic factor for monocytes and induces epigenetic modifications that exacerbate proinflammatory cytokine production [10, 11]. Also, PPAR-γ activation by the oxidized lipid fraction leads to differentiation into macrophages where oxLDL-stimulated macrophages are prone to migrate through a mechanism dependent on intracellular nitrosative stress and lipid peroxidation favoring their accumulation in the plaque [12, 13]. Moreover, since modified lipoproteins affect vascular cells as well, it has been found that endothelial cells increase their level of intracellular oxidative stress without oxLDL internalization due to the formation of reactive oxygen species (ROS) that permeate through the cell membrane [14]. Additionally, proinflammatory cytokines stimulate the proliferation and migration of smooth muscle cells, confining foam cells within fibrous tissue through mechanisms dependent on MAPK and NF-KB signaling [15], where a cytokine such as osteopontin involved in inflammatory and calcification processes promotes atheroma growth [16, 17]. In this way, ROS and reactive nitrogen species (RNS) present in the vascular environment create a pro-atherogenic condition exacerbated by the presence of an impaired equilibrium between the oxidizing and reducing capacity of the cell, a state known as oxidative stress.

Thus, the importance of oxidative stress in the development of atherosclerosis also relies on cellular responses triggered by an inadequate equilibrium between oxidants and reductants at different layers of the vascular tissue [18]. The diverse oxidation-reduction reactions that take place in this tissue consist of a strict exchange of electrons between molecules generating specific electric potentials (E°′) that can be modified by diverse tissue conditions. The electric potential will be greater for compounds prone to be reduced, where the most favorable reaction occurs between the strongest oxidant and the strongest reductant present in the reaction mixture [19], mostly mediated by ROS and RNS [20]. Although ROS and RNS present the capacity to oxidize many biomolecules, reductant molecules present in the microenvironment present the property to counteract the process. A strong oxidant such as the hydroxyl-radical might react with a strong reductant such as ascorbic acid, preventing an unwanted oxidation process and therefore becoming an “antioxidant,” a bioavailable reductant molecule that could prevent the progress of oxidative stress [21].

**Fig. 1:**
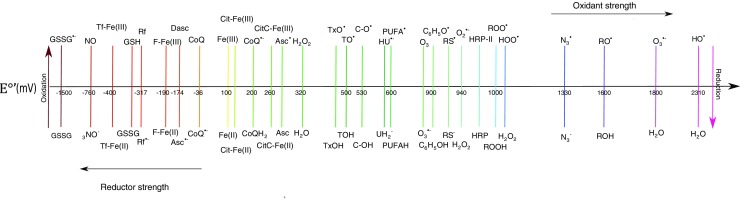
Redox potentials of biochemically relevant chemical species. Glutathione (GSSG), transferrin (Tf), riboflavin (Rf), ferritin (F), coenzyme Q (CoQ), citrate (Cit), cytochrome C (CitC), ascorbate (Asc), trolox (TxOH), tocopherol (TOH), catechol (C), uric acid(UH_2_^−^), polyunsaturated fatty acid (PUFA H2), free cysteine from protein (RS), horseradish peroxidase (HRP) [18, 25, 26]

While the difference in potential provides information about the physicochemical characteristics of the reaction, it is still dependent on the concentration of reactants as well as on cellular location. Although ascorbic acid (E°′ = 282 mV) is a stronger reductant than α-tocopherol (E°′ = 500 mV), the hydrophobic nature of tocopherol increases its antioxidant capacity, for instance against LDL-lipoperoxide formation, reason why tocopherol has attracted so much attention as an antioxidant in clinical research [22]. Since oxidation of lipids in plasma only occurs when ascorbic acid and α-tocopherol are, in turn, also oxidized [23], in a biological context, redox reactions depend on factors such as affinity of these molecules for lipoproteins, membranes, and/or diverse cellular compartments [24] (Fig. 1). Although the clinical approach for the use of antioxidant treatment is well supported and in some cases effective, this kind of supplementation still presents too many variables that require extensive analysis to conclusively prove its effect upon the process of atherogenesis in order to be thoroughly used in a clinical setting.

In this sense, it is interesting to mention that several years ago, Tsimikas et al. reported levels of oxidized LDL and Lp(a) lipoprotein in a total of 504 patients immediately before coronary angiography was carried out [27]. Interestingly, in the entire group of patients studied, the association between obstructive coronary artery disease and the ratio between oxidized phospholipid/apoB was independent of all lipid measurements and clinical condition except for Lp(a) lipoprotein. With these results in hand, they were able to conclude that circulating levels of oxidized LDL are strongly correlated in patients who present the diagnosis of coronary artery disease angiographically supported [27]. Overall, this kind of clinical data in great measure support observations and the early proposal made by JL Witztum focusing on the role oxidized LDL particles play during the process of atherogenesis [7].

## Vitamins and Antioxidant Molecules

The oxidative balance of the endothelial cell is highly related to the metabolism of lipoproteins and therefore to the development of atheroma lesions. In this regard, patients hospitalized for an acute myocardial infarction and, in general, populations at high cardiovascular risk tend to present a low non-enzymatic antioxidant capacity [28] and therefore a low plasma concentration of antioxidants [29, 30]. Epidemiological evidence suggests that plasma concentrations of different antioxidant compounds show an inverse relationship between the seriousness of the atherosclerotic process and its clinical manifestations, supporting the atheroprotective properties of several antioxidants [29].

Carotenoids correspond to a series of compounds synthesized by plants with redox potentials ranging from 980 to 1060 mV [30] and considered as weak reductants. Interestingly, populations showing high plasma concentrations of carotenoids, including cryptoxanthin, lycopene, and α-carotene, present a lower intima-media thickness than subjects with low plasma concentrations of these compounds [31]. In addition, α- and β-carotene concentrations have shown an inverse association with atherosclerosis when the presence of plaque in the carotid and femoral arteries was evaluated by ultrasound [32]. Also, when β-carotene negatively correlates with interleukin-6, the inflammatory process is favored [33]. Nevertheless, despite the evidence regarding the correlation between the protective effect of carotenoids and the presence of a lower cardiovascular risk, statistical significance of most results has not been maintained after correction for the presence of risk factors such as high blood pressure and high cholesterol levels [34]. Similarly, no statistically significant results were found in a population of 22,000 men receiving for 12 years an oral administration of β-carotene [35]. Among carotenoids, lycopene has been identified to be the compound with the highest reducing capacity tested in reactions involving a singlet oxygen (650 mV) [36]. This reducing capacity has aroused interest related to atherosclerosis; however, its activity has only shown to reduce LDL oxidation in vitro [37], but not lipid peroxidation or LDL oxidation in vivo [38].

According to ultrasonic evidence, asymptomatic patients that show the presence of plaque in the carotid arteries in comparison to patients with normal arteries present low plasma concentrations of lycopene [39]. These results are supported by the fact that lycopene administration along with other antioxidants drastically reduces atherosclerotic plaque in transgenic mice models [40] and in healthy volunteers improves endothelial function [41, 42]. Among these properties, lycopene promoted the metabolism of lipids through changes in protein expression in both in vitro and in vivo models [43]. Although these results suggest that lycopene presents a strong atheroprotective capacity due to its diverse biological activities, it is still necessary to understand the specific metabolic pathways that become modified in order to fully explain its molecular effects [44–46].

Since strong reductants are expected to generate better clinical outcomes, new research employing tocopherol, presenting a protective role for LDL oxidation, has been launched with its natural source, vitamin E, together with several chemically derived molecules [23, 47–51]. In this respect, although the presence of vitamin E in plasma shows an inverse correlation with the development of ischemic heart disease [49] and its consumption is associated with a lower incidence of CAD [50], it has been observed that the molecule is oxidized in plasma before LDL particles are affected when synthetically oxidizing systems are employed [23]. The administration of α-tocopherol produced a dramatic decrease (77%) in the risk of non-fatal myocardial infarction in a population with a clinical history and angiographic evidence of coronary atherosclerosis [45]. Nevertheless, according to the α-tocopherol, β-carotene supplementation on coronary heart disease (*ATBC*) study, the molecule marginally decreased the incidence of major coronary events and fatal coronary heart disease (4 and 8%, respectively) [51]. The ATBC cohort monitored for 6 years showed that at the end of the clinical trial, the incidence of a first-ever major coronary event non-fatal myocardial infarction, or fatal coronary artery disease, did not decrease. Nevertheless, the follow up of patients 2 years after the completion of this study showed a significant decrease in incidence rates but was not further analyzed [52]. Several other trials have shown results that were not equally encouraging since treatment with vitamin E in a population with high cardiovascular risk and a history of cardiovascular disease, diabetes, and other risk factors did not reduce the prevalence of cardiovascular-associated deaths [53] even in combination with vitamin C, β-carotene, or both [54–59].

Ascorbic acid can be considered a reductant of average strength with respect to the range of chemical species present in plasma. Induced atherosclerosis in animal models, either by promoting changes in cholesterol metabolism or by oxidative stress, showed up to half the size of plaque in a vitamin C-administered group [55, 56]. Although it is hydrophilic in nature, ascorbic acid reduced up to 60% the oxidation of LDL by myeloperoxidase in contrast to the lack of effects produced by tocopherol [54] at plasma concentrations [58, 59]. On the other hand, co-administration of vitamins in experimental atherosclerosis produced superior positive changes in the size of the atherosclerotic plaque and in the presence of oxidation markers in comparison to treatment with each of the antioxidants separately [55]. Diverse clinical trials performed in different populations presenting a high ascorbic acid plasma concentration showed a low prevalence of angina [58]. Moreover, the administration of large doses was associated with a reduced cardiovascular risk [60] in support of studies showing that low plasma ascorbic acid concentrations are associated with a higher incidence of myocardial infarction [61]. A meta-analysis of 44 clinical trials showed a relationship between the administration of doses over 500 mg/day of vitamin C and an improvement in endothelial function for patients with diabetes, atherosclerosis, and heart failure, despite the fact that no improvement was found in healthy volunteers [62]. However, in a more ambitious approach, administration of ascorbic acid to 70-year-old patients did not diminish the incidence of deaths due to cardiovascular disease [54] nor did modify the conventional parameters of cardiovascular risk, such as markers for oxidative stress [63].

The sum of these results thus far, although not conclusive regarding the potential benefits of antioxidant therapies, might be related to the fact that the methodology employed is not the same in every trial, a situation that will have to be further investigated in the near future. Several of these studies do not allow detecting the level of protection because specific concentrations of antioxidants in plasma were not reported. Additionally, several epidemiological studies lack the analysis of the initial conditions of the total antioxidant capacity of plasmas, since the same benefit is not necessarily provided to all individuals with the same antioxidant dose when the benefit due to the administration of antioxidants was recorded only when there was a prior deficiency [64]. It is feasible to suppose that not all people are subjected to the same oxidative stress, that not all people have the same antioxidant defenses, and that they all do not have the same response to the same treatment. Since as shown in several studies these variables have not been considered in the design and analysis of the clinical trials, this may be one of the reasons why results range from a 77% reduction to a lack of effectiveness when studying the incidence of myocardial infarction. In addition, an important premise in several of these trials has been forgotten as oxidative stress seems to be a crucial factor during atherogenesis and less critical when lesions are well established. Studies employing experimental atherosclerosis show that although the administration of antioxidants simultaneously to an atherogenic stimulus reduces the number of atherosclerotic lesions, in the clinic, the use of antioxidants has been mostly limited to populations of advanced age, omitting that the process of atherogenesis in humans may start before birth [65].

Due to the significant uncertainty surrounding the use of antioxidants, treatment with stronger reductant molecules chemically derived from tocopherol has been attempted with promising results. The case of probucol is extremely interesting since its antioxidant capacity has been shown to be superior to that of the parent compound, reducing the atherosclerotic plaque from 54 to 7% and considerably reducing the ex vivo oxidation of LDL in animal models [66, 67]. High-cardiovascular-risk populations with atherosclerotic plaque confirmed by angiography showed a drastic reduction in high-density lipoprotein-associated cholesterol (HDL-C), an increase in the QTc interval, and a reduction in lumen volume from the start of a 3-year treatment with probucol and cholestyramine [68]. In consideration that lumen volume is not a clear reflection of the presence or severity of the atherosclerotic plaque, a study was conducted in hypercholesterolemic patients focusing on the thickness of the intima-media layer which was reduced by 14% in 2 years under probucol treatment [69]. Nevertheless, due to the importance attributed to maintaining the proper cholesterol metabolism during treatment, a decrease in HDL-C was a relevant factor against the use of probucol, which led to the search for other chemically synthesized antioxidants that would not adversely impact the normal lipid profile. The substitution of functional groups in one of probucol phenolic rings yielded the synthesis of succinobucol, an antioxidant compound that is not susceptible to modification by metabolism, thus preventing the formation of the hepatotoxic molecule spiroquinone and showing an anti-inflammatory action through binding to VCAM-1 [70]. Although succinobucol inhibited the development of atherosclerosis in different animal models and selectively decreased LDL-C and increased HDL-C [71], healthy subjects treated with succinobucol showed a decrease in HDL-C and apo AI concentrations, changes associated with an increase in LDL-C mainly through changes in the LDL3 subclass. These changes were also significantly correlated with an increase in plasma CETP mass. Moreover, administration of succinobucol to patients subjected to a percutaneous coronary intervention did not produce changes in the volume of plaque measured by intravascular ultrasound compared to placebo [72], while patients with prior acute coronary syndromes showed a lower incidence of myocardial infarction or cerebrovascular events [73]. Interestingly, a prolonged release of succinobucol studied in pigs using a metallic stent induced inflammation and tissue deterioration interfering with the healing process of blood vessels [74]. These disorders may be largely due to the inhibition of smooth muscle cell proliferation and also by an induced cell apoptosis allowing an increment in mitochondrial ROS through cytochrome c peroxidase activity, all of which prevent revascularization [75].

On the other hand, the hydrophobic antioxidant BO-653 inhibited LDL oxidation and the development of atherosclerotic lesions in different animal models compared to the effect of probucol [76]. Rabbits with vascular damage induced by denudation of the iliac artery under a cholesterol-enriched diet presented an effect upon c-myc, one of the cell cycle main controls, suggesting that the clinical use of probucol may compromise re-endothelialization [77]. By comparison, administration of elsibucol to rabbits fed with a cholesterol-rich diet improved significantly IM thickness even in comparison with probucol. Unlike succinobucol, elsibucol-inhibited VSMC proliferation evens at concentrations four-times higher than those found in plasma, demonstrating also a level of re-endothelialization comparable to controls. Nevertheless, cholesterol metabolism showed some impairment since HDL-C was decreased with no apparent effect upon LDL-C. Although this could be considered a negative effect, it is necessary to assess the mechanisms of modification of lipoproteins in order to know whether this result corresponds to a retention of particles or to a more efficient elimination of excess lipids that could lead to a metabolic improvement [78, 79].

There have been some other attempts to control atherosclerosis with antioxidants from natural sources, like the study from Shen et al. showing that treatment of atherosclerotic mice with quercetin induces the expression of heme-oxygenase-1 and promotes a reduction in oxidative stress markers attenuating endothelial dysfunction induced by a lipid-rich diet or even strong oxidants [80, 81]. Surprisingly, all these benefits were obtained with a dosage close to the average daily intake reaching a concentration level well below its detection limit in plasma when employing highly sensitive techniques such as HPLC coupled with mass spectrometry [80]. Furthermore, the administration of quercetin in LDLR−/− mice fed with an atherogenic diet and subjected to an exercise routine showed an enhancement of LDL resistance to oxidative modification and a reduction of plaque [81]. Quercetin administration associated with exercise showed an important increase in the hepatic expression of ABCA-1, apo A-4, and PPAR-α. However, concomitantly, there was a decrease in apo AI gene expression, the main protein component of the widely considered anti-atherogenic high-density lipoproteins, which resulted in a point against a daily use of quercetin [82]. Associated with the effects shown by quercetin, polyphenolic extracts obtained from grape seeds containing compounds such as hydroxycinnamic acid, flavonols, and stilbenes reduced intracellular oxidative stress through a lower expression of VCAM-1, ICAM-1, E-Selectin, MCP-1, and M-CSF that inhibited the binding of macrophages to endothelial cells activated by exposure to lipopolysaccharides [83]. Changes in protein expression were primarily due to the activation of the transcription factors NF-Κβ and AP-1 by stimulation with lipopolysaccharides. Both factors are characteristic of systems presenting oxidative stress associated with several inflammatory responses and their activation [84–86].

Therefore, the statistical correlation between plasma concentrations and the atherosclerotic status of patients together with the existing evidence for the potential antioxidant atheroprotective role of several molecules represents a very encouraging scenario to search for new molecules. We do believe it is important to highlight that natural extracts, containing low concentrations of active molecules and therefore difficult to measure since they are present below their detection range, by exerting a synergistic action might modify atherogenic patterns. This approach can give a new direction to the search of potential antioxidant therapies that might modify the process of atherogenesis, not as single molecules but as a synergistically protective natural mixture.

Although oxidation-reduction reactions play a crucial role in atherogenesis, analyzing antioxidants only with regard to their reduction potential might be a too narrow perspective. For instance, *Perilla frutescens* extracts and one of its component, α-asarone, showing a strong antioxidant capacity prevent the oxidation of LDL in vitro and in vivo [87]. α-Asarone potentiated the macrophage response to LXR and PPAR-γ agonists, diminishing SR-B1 and increasing the expression of ABCA-1 and ABCG-1 and therefore explaining its effect through an antioxidant activity and the modulation of lipid metabolism [88]. Another example is sesame oil, a mixture rich in lignans and other antioxidants that, when tested in LDLR^−/−^ mice fed with an atherogenic diet, significantly inhibited atherosclerotic plaque development, mediated partially by a favorable impact on lipid profiles [89]. A sesame oil aqueous extract increased the lag time in conjugated diene formation during LDL and HDL oxidation by exposure to Cu^2+^ and MPO activity [90]. Employing macrophages stimulated with lipopolysaccharides and endothelial cells treated with TNF-α and moderately oxidized LDL, the extract inhibited transcription and synthesis of proinflammatory cytokines IL-6, Il-1a, TNF-α, chemokines, adhesins MCP-1, and VCAM1, in addition to inhibiting SR-A1 and inducing ABCA-1 involved with cholesterol exchange between cells and lipoproteins. This type of gene expression is triggered by oxidative stress-sensitive transcription factors and ligands affecting LXRs activation and NF-Κβ inhibition and translocation [91]. Such biochemical responses could result from just one single multifunctional antioxidant, or rather due to advantages from several antioxidant compounds within the extract that might have been potentiated among them.

Although it can be said that there is important evidence for the potential use of antioxidants as atheroprotective molecules in the clinic, the only way to fully support this statement will be to importantly improve the way to standardize their effects directly correlating their physiological effects with their concentration in plasma. Moreover, in the case of studying as a source for antioxidants the use of natural extracts, the way to standardize their anti-atherogenic effects will be only achieved by also investigating their synergistically active components and the way these components when present in plasma below a detection value can provide a positive atheroprotective effect.

## Regulation of Protein Expression

Compounds and molecules with a reducing ability not only prevent physiological deterioration of vascular cells and lipid peroxidation of LDL but can also promote or inhibit the expression of proteins that present an atheroprotective effect. High-density lipoproteins exhibit atheroprotective characteristics that include mobilization of excess cholesterol to the liver and an antioxidant activity associated with paraoxonase-1 (PON1) [92–94]. PON1 hydrolyzes lipid oxidation products, prevents their formation reducing intracellular stress of macrophages in vivo [94], and presents a reduction capacity that has been shown to be diminished in plasma from patients with a recent myocardial infarction and those at high cardiovascular risk [95]. Another characteristic of this enzyme involves the improvement of cholesterol efflux by macrophage ABCA-1 expression promoting binding through its amphipathic helices with membrane cholesterol lipid rafts [96] and a catalytic core composed of glutamic acid, asparagine, and aspartic acid with activity dependent on free thiols [97, 98].

On the other hand, it has been described that after delipidation, HDL particles show an antioxidant capacity independent from PON1 [99]. In this respect, Kotosai et al. showed that apo AI reacts selectively with free fatty acid hydroperoxides in a mechanism dependent on methionines, reducing these compounds to stable oxidation products such as fatty acid hydroxyls [100]. Other apolipoproteins associated preferentially with HDL effectively bind oxidized LDL phospholipids, reducing their rate of oxidation and increasing the lag time for the process to take place [101]. In general, cellular responses to modified protein structures could be an essential step to understand pathogenesis and therefore the atherogenic processes [102].

Since proteins account for approximately 70% of the dry cell mass, assays conducted on cells under constant generation of hydroxyl radicals (E°′ = 2310 mV) show protein peroxides as the main oxidized product with almost nonexistent lipid and nucleic acids oxidation products [103] and where the generation of protein peroxyl radicals weakens intracellular antioxidant defenses [104]. In this regard, the efficient defense system shown by ascorbic acid seems to reside in both preventing the formation of peroxyl radicals and the acceleration of the decay process of these radicals to stable hydroxide species [105, 106]. Ascorbate also protects the intracellular levels of glutathione, where both antioxidants combined prevent further intracellular protein peroxide formation [107]. Considering glutathione as a strong reductant (E°′ = − 1500 mV), it is to be expected that its presence in plasma attenuates early vascular lesion development as observed in a hyperlipidemic mice model [108, 109]. It has been observed that glutathione plasma concentration seems to be a critical factor in the development of the atherosclerotic plaque since an 80% decrease in its intracellular concentration promotes the development of complex vascular lesions [103, 109]. This information is further supported by experiments where bone marrow transplantation in experimental animals capable of synthesizing up to three-times more glutathione than normal, reduced the progression rate of vascular lesions by approximately 35% [103].

Several models for atherosclerosis such as transgenic mice with humanized and pro-atherogenic lipid metabolism have shown by the administration of ribose-cysteine an increased glutathione and glutathione peroxidase activity in liver tissue and plasma. This modification appears to be responsible for a reduced content of oxidized biomolecules in the liver, plasma, and aorta, in addition to a reduction in LDL-C, apoB, Lp(a), and total cholesterol plasma concentration associated with an increase in LDLR expression [110].

Both the concentration and activity of proteins are altered through oxidative modification, regulation of expression, and post-transcriptional silencing of non-coding RNA fragments (micro RNA or miRNA) paired to the 3′ untranslated regions from target genes [105].The human genome encodes approximately 1800 miRNAs capable of regulating protein function through direct binding to their 3′UTR [106] and by several indirect mechanisms through regulation of repressors or transcription factors [111]. Therefore, miRNAs have proven to impact the development of atherosclerosis, for instance, inhibiting LDLR and ABCA1 expression in hepatic cells [112]. Overexpression of miRNA-223 down-regulated SRBI and HMGCS limiting cholesterol synthesis and indirectly increasing ABCA1 due to the modulation of transcription factor Sp1 [113]. In hepatocyte cell cultures, miRNA-27a decreased by 40% the levels of LDLR increasing the concentration of PCSK9, which enhances LDLR degradation and regulates LRP6 and LDLRAP1 [114]. Results from in vivo models provide an even more exciting scenario since stable atherosclerotic lesions show different patterns for miRNAs in comparison to complex lesions prone to rupture, where inhibition of miRNA-494 led to a decrease of plaque size and the presence of more stable lesions [115].

However, the inhibition of specific miRNAs in cell culture cannot be easily extrapolated to a therapeutic approach since the presence of miRNAs in circulation might affect not only the vascular tissue but also other organs and cell systems while being transported by HDL [116, 117]. Proteins such as PON1 with intrinsic antioxidant capacity are also regulated by specific miRNAs [118]. On the other hand, several antioxidant compounds such as resveratrol modulate the expression patterns of several miRNAs involved in both cancerogenic and inflammatory processes [119]. A clinical trial involving male patients suffering from hypertension with associated diabetes mellitus type2 and coronary artery disease, after 1 year of an 8-mg daily intake of resveratrol, revealed a decrease in the expression of proinflammatory cytokines modifying the level of several miRNAs [120]. Orally administrated polyphenols to hyperlipidemic mice also prevents fatty liver disease through the action of miRNA103 and miRNA122 [121], whereas catechins are capable of changing the expression of hepatic miRNAs in apoE-deficient mice and HepG2 cells [122].

All this evidence lead us to further evaluate the potential effects of miRNAs as key regulators of many antioxidant proteins involved in the defense mechanisms against ROS generation. Among all miRNAs that might be modulated by antioxidant compounds, we need to keep in mind that exogenous miRNAs can be absorbed from the diet, and therefore, found in plasma where they could also modify gene expression [123]. Zhang et al. have proven that miRNAs derived from plants can be absorbed in the gastrointestinal tract reaching the plasma in stable microvesicles showing an effect upon LDLRAP1 [124].

## Perspectives for Antioxidant Therapy

To date, accumulated evidence regarding oxidation as a pro-atherogenic factor indicates that biochemical events involved in this process are indeed a very attractive target for the management of cardiovascular disease in the clinic. Nevertheless, although evidence indicates that redox reactions are important in the initiation and progression of atherosclerosis, oxidation with a pro-atherogenic context does not eliminate the fact that oxidation participates in many cases as an essential messenger of cellular signaling pathways [125]. Therefore, disease management and therapeutic goals require not only high-precision and high-sensitivity methods to detect in plasma very low amounts of reducing and oxidizing molecules but also a much better understanding of the normal processes and metabolic pathways influenced and/or controlled by oxidative stress. As several methodologies have been specifically described for the quantitation of the total antioxidant capacity and the oxidation state of diverse biological systems [25, 26], a successful way to carefully study how redox reactions influence atherosclerosis can be achieved. Nevertheless, since there is still a lack of standardization in many of these methods, clinical trials studying antioxidant capacity have been difficult to compare and therefore difficult to use in order to reach a conclusion. On the other hand, there is also a problem to consider related to the possibility that in many of the studies discussed in this review, the concentration of the specific antioxidant did not reach the critical active concentration at key sites known to be important in the development of atherosclerosis, for instance the intima of blood vessels or the hepatocyte. This is an important point that will have to be technically solved in order to have the certainty that future clinical trials will present the possibility to directly correlate specific tissue concentration of antioxidants and prevention with the development of the disease.

**Fig. 2:**
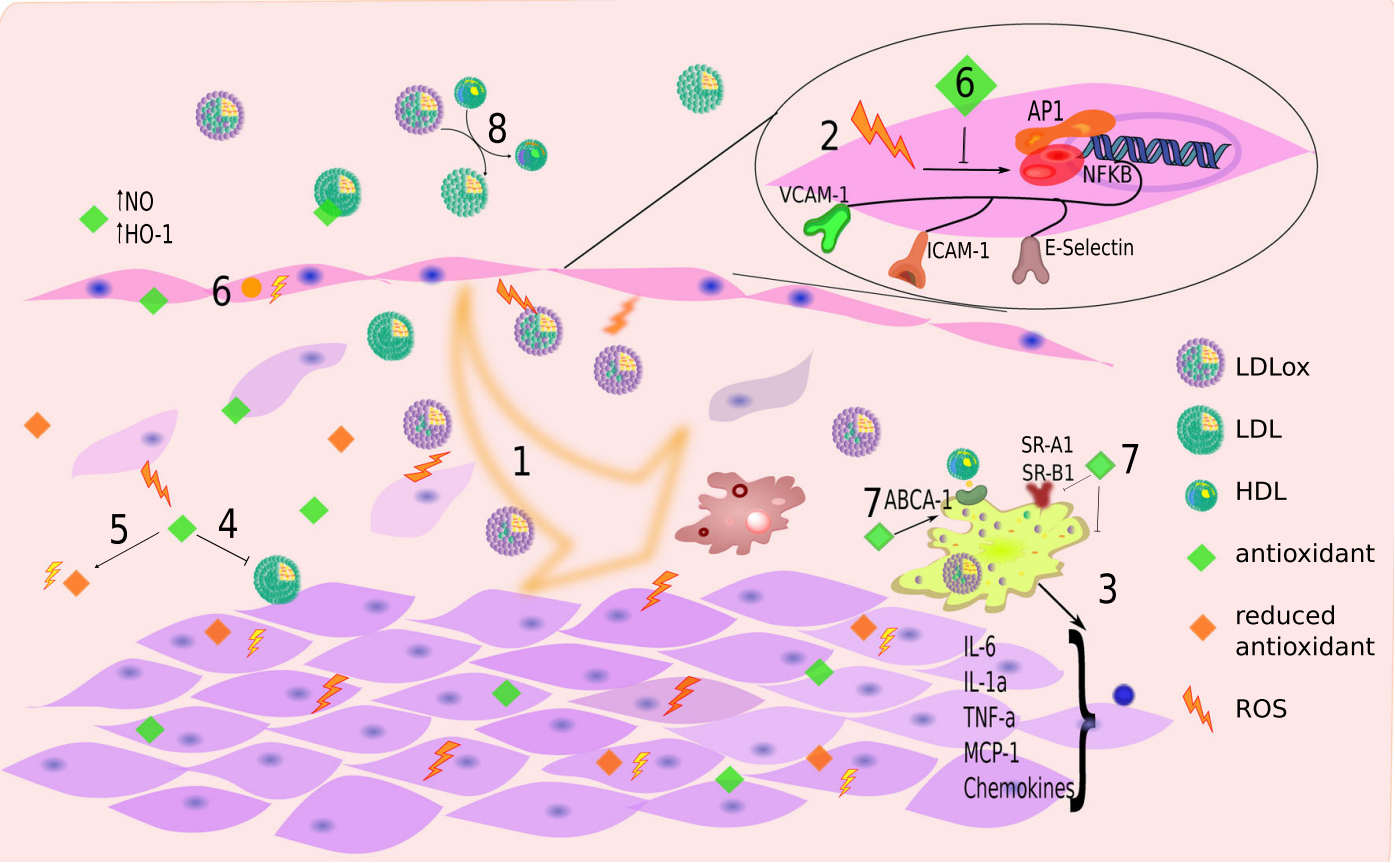
Interaction between ROS and antioxidants. Since LDL particles present in the subendothelial space constantly remain exposed to ROS, lipoproteins gradually become transformed into oxidized particles (oxLDL) (1). Cells exposed to oxLDL activate transcriptional factors and several receptor expression patterns leading to cell metabolism changes (2). Phagocytosis of oxLDL carried out by macrophages promotes their transformation into foam cells prone to release intercellular signaling molecules that favor inflammation (3). Nevertheless, the presence of antioxidant molecules in the subendothelial space may avoid LDL oxidation (4). Antioxidants react with reactive oxygen species (ROS) (5), exerting a protective role against cellular damage due to oxLDL formation (6). Antioxidants might enhance or diminish the presence of specific receptors and intracellular enzymes that in general promote the presence of an anti-atherogenic metabolic status (7). Despite oxidation, interaction with HDL can regenerate LDL from oxLDL (8)

Since we have recently found a direct relationship between the exposure of not only chemically modified LDL but also normal LDL going through a “normal” process of oxidation with specific transcriptomic changes in vascular smooth muscle cells [126], several antioxidant molecules support their activity by creating a proteomic or even a miRNA pattern that leads cell expression to what can be recognized as an antioxidant metabolism pattern (Fig. 2). Nowadays, a broad spectrum of tools including proteomic, metabolomic, and transcriptomic approaches have been developed to render detailed information related to the many changes found in the metabolism of cells due to a specific antioxidant treatment [127–130]. Therefore, challenging research providing brand new data and more importantly brand new concepts is ahead of us. For instance, to find an efficient way to target antioxidants to specific intracellular organelles such as lysosomes and mitochondria, or to study subtle changes that might occur in the epigenetic control of gene expression secondary to antioxidant therapy, could be considered as two interesting approaches. We believe a comprehensive analysis of this new knowledge and its relationship with the presence of plasma antioxidants and their reducing capacity will undoubtedly open new ways to understand and develop new therapeutic pathways in the fight not only against atherosclerosis but also against other degenerative diseases.
